# Profiles of Basic Psychological Needs in Exercise Settings: An Examination of Differences in Contextual Motivation, Affect, and Achievement Goals

**DOI:** 10.3390/ijerph15122871

**Published:** 2018-12-14

**Authors:** Zişan Kazak

**Affiliations:** Faculty of Sport Sciences, Ege University, Bornova, 35100 Izmir, Turkey; f.zisan.kazak@ege.edu.tr; Tel.: +90-232-342-57-14

**Keywords:** exercise, cluster analysis, basic needs, motivation, affect

## Abstract

Understanding leisure-time physical activity is vital for a healthy world. It is well known that physical activity has positive effects on psychological health, but further evidence is required to ascertain how different environments influence positive behavioral outcomes. Thus, the aim of this study was to examine the differences in contextual motivation, affect, and achievement goals according to profiles of basic psychological needs in adult exercisers. The sample consisted of 401 young adult exercisers ranging in age from 18 to 40 years from fitness centers in Izmir, Turkey. Participants completed measures of basic needs satisfaction, exercise motivations, trait affect, and achievement goals. Hierarchical cluster analysis, k-mean cluster analysis, multivariate analysis of variance, and post hoc analyses were performed. The results in this study revealed that the participants in Cluster 1, who were labeled as “very satisfied”, had higher scores than the other clusters in terms of positive behavioral outcomes. The results of this study revealed that greater satisfaction of the basic psychological needs leads to positive behavioral consequences in exercise contexts.

## 1. Introduction

Self-determination theory (SDT) is a macro theory about the influence of personality development, basic psychological needs, subjective well-being, and social environment on affect, behavior, and motivation [1]. SDT consists of several mini-theories. At the center of each mini-theory are the basic psychological needs of autonomy, competence, and relatedness. All individuals strive for these needs in order to flourish and grow [2]. Autonomy is a term that refers to regulation by the self [3]. The need for autonomy refers to individuals choosing to initiate their own actions, to make their own choices, and to experience self-direction [2,4,5]. The need for competence concerns people’s need to feel competent and a sense of mastery in interacting with their environment [6,7]. The need for relatedness involves a need to experience a sense of belonging and attachment with other people [8]. According to Deci and Ryan [8], these needs are the universal and innate basic elements necessary for survival, healthy development, integration, and psychological well-being. SDT is generally centered on these three needs and their essential role in self-determined motivation.

In satisfying the basic psychological needs, social environment, relations, and culture are as important as the personality because all lives and behaviors are influenced by social context [9]. In other words, the satisfaction of needs is a dynamic process that is influenced by the individual, social environment, and culture. Wilson et al. [10] indicated that participating in sports and exercise settings could be promoted as a means of satisfying individuals’ psychological needs. The satisfaction of individuals’ psychological needs is also assumed to lead to more autonomous forms of regulation and to enhance psychological and physical well-being and feelings of vitality [1,8,11]. Basic needs are related to positive and negative affect, which are the affective components of subjective well-being. Some researchers [12,13,14] have reported in their studies that psychological need satisfaction was positively related to positive affect and negatively related to negative affect. In the study of Podlog and his colleagues [15], it was reported that competence and autonomy were associated positively with positive affect. Also, in the same research, it was determined that relatedness was associated negatively with negative affect. 

Previous research showed that satisfaction of psychological needs through physical activity is related to more self-determined motivation [16,17]. Intrinsic motivation is the most self-determined form of motivation. According to SDT, intrinsic motivation can be promoted or thwarted according to the degree of fulfillment of three basic psychological needs [18]. The need for relatedness is also a very important need for intrinsic motivation. Intrinsic motivation is more likely to develop in contexts in which the sense of close relationship with others is experienced. If needs for competence, autonomy, and relatedness are supported by the social environment, initially extrinsically motivated behaviors can become internalized, and more self-determined forms of extrinsic motivation may result [19]. In Deci, Ryan, and Williams’ study [20], it has been stated that satisfaction of the needs in a learning environment provides development of intrinsic motivation and facilitates internalization of extrinsic motivation. 

The relationship between self-determination theory and achievement goal theory has been revealed in many studies (e.g., [21,22,23]). These two theories both emphasize the role of autonomy and perceived competence [8,24]. The individual components of self-determination theory (i.e., competence, autonomy, and relatedness) are in congruence with some of the major antecedents of achievement goals. Achievement goal theory focuses on successful behavior and competence, and it states that competence plays a large role in the constructs related to achievement goals, and it can be differentiated along two basic dimensions according to how it is defined and valenced [25]: mastery (that is, task goals or learning goals) and performance (i.e., ego goals). These constructs are reorganized as the 2 × 2 achievement goal framework by Elliot [25]. Elliot and colleagues [26,27] have argued that the dichotomy between mastery and performance goal orientations should be revised to include the distinction between approach and avoidance motivations. Elliot and McGregor [28] developed four types of goals by combining mastery and performance dimensions with approach and avoidance dimensions: mastery-approach goal, mastery-avoidance goal, performance-approach goal, and performance-avoidance goal. Individuals with mastery-approach goals have absolute standards, focus on intrapersonal competence, and strive to perform better than one has done before, to increase their competence, and to master a skill [29,30]. The mastery-avoidance goal avoids task-based or intrapersonal incompetence by striving to not perform a skill more poorly than before [28,30]. The performance-approach goal defines a desire to attain normative competence by way of doing better than others, whereas the performance-avoidance goal defines a desire to avoid normative incompetence by way of doing worse than others [31]. As is clear from the definitions of the four achievement goals, perceived competence plays an essential role in the four achievement goals. Diseth and colleague [32] indicated that the need for competence predicted mastery achievement goal both directly and indirectly, and basic need support is obviously more important for mastery than for performance goals.

The purpose of the present study was to examine contextual motivation, affect, and the 2 × 2 achievement goals according to profiles of basic psychological needs in exercise settings. Based on the tenets of SDT and past research, it was hypothesized that levels of contextual motivation, negative and positive affect, and the four achievement goals in exercise settings would differ according to the level of satisfaction of the three basic psychological needs considered in this study. 

## 2. Materials and Method

### 2.1. Participants

The sample consisted of 401 adult exercisers attending different sport centers in Izmir, Turkey (*n* = 193 females and *n* = 208 males). Participants ranged in age from 18 to 40 years (*M* = 26.16, *SD* = 5.98). The participants self-reported as representing various exercise groups (general fitness, Pilates, swimming, TRX, aerobic, walking, Zumba, running, kickboxing). The participants, on average, self-reported having a healthy body mass index (BMI) of 23.00 ± 3.07 kg/m^2^ and mean (*M*) = 271.93 (*SD* = 158.21) minutes per week of intentional physical activity and approximately seven years of sports experiences (*M* = 6.48, *SD* = 6.08 years).

### 2.2. Measures

#### 2.2.1. Achievement Goals 

The 2 × 2 Achievement Goals Questionnaire for Sport (2 × 2 AGQ-S) [33] assessed exercisers’ achievement goals. The questionnaire consists of 12 items and the following four subscales: mastery-approach (MAp), mastery-avoidance (MAv), performance-approach (PAp), and performance-avoidance (PAv). Participants were presented with a common stem in the questionnaire: “At the sports center…”. Responses were provided using a seven-point Likert-type scale from 1 (strongly disagree) to 7 (strongly agree). Scores from the Turkish version of the 2 × 2 AGQ-S have demonstrated acceptable reliability and validity [34]. In the present study, Cronbach’s α coefficients were 0.89 for mastery-approach, 0.77 for mastery-avoidance, 0.88 for performance-approach, and 0.82 for performance-avoidance. 

#### 2.2.2. Basic Needs 

The Turkish version [35] of the Basic Psychological Needs in Exercise Scale (BPNES) [36] was used to determine the basic psychological needs of the individuals in exercise settings. The scale has 12 items and three subscales: autonomy, competence, and relatedness. Participants rated each item on a five-point Likert scale ranging from 1 (strongly disagree) to 5 (strongly agree). Higher scores demonstrate that participants consider their needs to be more fulfilled. In this study, the internal consistency coefficients for the BPNES subscales were 0.74 for autonomy, 0.80 for competence, and 0.79 for relatedness.

#### 2.2.3. Exercise Motivation 

The participants completed the Behavioural Regulation in Exercise Questionnaire-2 (BREQ-2) [37] to assess their contextual motivation levels. This scale consists of 19 items and five subcategories (intrinsic motivation, identified regulation, introjected regulation, external regulation, and amotivation) on a five-point scale ranging from 0 (not true for me) to 4 (very true for me). The BREQ-2 has been shown to have good factorial validity (e.g., [38,39]). In the present study, Cronbach’s α coefficients were 0.77 for intrinsic motivation, 0.60 for identified regulation, 0.75 for introjected regulation, 0.61 for external regulation, and 0.62 for amotivation. 

#### 2.2.4. Affect 

Positive affect (PA) and negative affect (NA) were measured via the Positive and Negative Affect Scale (PANAS) [40]. Gençöz [41] translated the PANAS into Turkish. The scale comprises 20 items divided into two subscales, with ten words per item making up the PA and NA subscales. Respondents were asked to rate the extent to which they felt each emotion in exercise contexts during the past two weeks. The responses were anchored on a scale of 1 (very slightly or not at all) to 5 (extremely). Scores can range from 10–50, and higher scores represent higher levels of positive and negative affect. The construct validity and internal reliability of the Turkish version of the PANAS have been successfully demonstrated [41]. In this study, Cronbach’s alpha values of 0.79 and 0.82 were obtained for negative and positive affect, respectively.

### 2.3. Procedure

The author obtained permission from the directors of a number of fitness centers. Then, potential participants were informed about the current study verbally in addition to being provided a written document by the author. All potential participants voluntarily filled out an informed consent to participate following the Declaration of Helsinki. Participants were encouraged to answer the questionnaire honestly. They were reminded that their answers would be confidential. They completed the questionnaire packet at the time of consent and prior to exercise on that day in a room with tables and chairs provided. This process took approximately 15 min. Data were collected across all days of the week. 

### 2.4. Data Analysis

Windows of statistical software IBM SPSS^®^ version 25.0 (IBM Corp, Armonk, NY, USA) was used to analyze data. Firstly, all data were checked for entry errors. Next, the scales were scored. Once the scales were scored, a check for any outliers occurred. Outliers were defined as standardized residual values above −3 and +3. Outlying values were deleted from the data. Secondly, a hierarchical cluster analysis using with Ward’s method was performed and the Euclidean squared distance was evaluated for the three basic needs to identify profiles. Correlations among all variables were calculated, and multivariate analysis of variance (MANOVA) was used to evaluate the basic need fulfillment profiles with other variables. The Ryan-Einot-Gabriel-Welsh Q (REGWQ) post hoc test was the follow-up test for potential univariate *F* differences. Partial eta-squared (η^2^) and Hedges’ *g* values were examined in order to interpret meaningfulness, and Cohen’s [42] interpretation guidelines were followed for effect sizes. All analyses were conducted using a significance level of 0.05. Data are reported as means and standard deviations.

## 3. Results

### 3.1. Descriptive Statistics and Correlational Analyses

Descriptive statistics and correlations among the variables in this study are presented in Table 1. These descriptive statistics for the entire sample revealed moderately high levels of MAv (*M* = 3.69; *SD* = 1.66) and PAv (*M* = 3.87; *SD* = 1.75) as well as for the introjected regulation (*M* = 3.12; *SD* = 1.04). In this sample, lower values were obtained for amotivation (*M* = 1.28; *SD* = 0.42), external regulation (*M* = 1.42; *SD* = 0.49), and PANAS PA (*M* = 18.07; *SD* = 6.15). MAp (*M* = 5.52; *SD* = 1.55), PAp (*M* = 4.36; *SD* = 1.83), identified regulation (*M* = 4.20; *SD* = 0.61), intrinsic motivation (*M* = 4.39; *SD* = 0.61), PANAS PA (*M* = 36.14; SD = 6.72), autonomy (*M* = 4.13; *SD* = 0.56), competence (*M* = 4.14; *SD* = 0.57), and relatedness (*M* = 4.06; *SD* = 0.63) were found to be high in this sample.

When the correlation results were examined (Table 1), it was revealed that the mastery-approach goal was correlated with all variables in this study. The mastery-avoidance goal was only related to external regulation and PANAS NA. As for the performance-approach goal, it was correlated with all of the other variables, except for external regulation and PANAS NA. On the other hand, the performance-avoidance goal was related to all of the other variables, expect amotivation and PANAS NA. PANAS NA was not related to external regulation, introjection regulation, identified regulation, and intrinsic regulation. Basic needs were related to all of the other variables, expect MAv.

### 3.2. Cluster Formation

The agglomeration schedule and the dendrogram indicated three clusters to be reasonable cluster solutions. After this analysis, the clusters obtained from the hierarchical analysis were validated with K-means cluster analysis. These clusters were examined and labeled. According to this, the first cluster was labeled as the “very satisfied” group, the second cluster as the “satisfied” group, and the third cluster as the “moderately satisfied” group. According to these results, three profiles emerged from the hierarchical cluster analysis that were all significantly different (Wilks’ λ = 0.75, *F*_(18, 780)_ = 6.67 *p* < 0.001, η^2^ = 0.13). There were 123 participants in the group labeled as the “very satisfied” who were characterized by high autonomy (*M* = 4.74, *SD* = 0.29), competence (*M* = 4.76, *SD* = 0.28), and relatedness (*M* = 4.76, *SD* = 0.31); 194 participants in the group labeled as “satisfied” who were characterized by high autonomy (*M* = 4.02, *SD* = 0.29), competence (*M* = 4.05, *SD* = 0.26), and relatedness (*M* = 3.96, *SD* = 0.32); and 84 participants in the group labeled as “moderately satisfied” who were characterized by high autonomy (*M* = 3.46, *SD* = 0.37), competence (*M* = 3.42, *SD* = 0.41), and relatedness (*M* = 3.29, *SD* = 0.39).

### 3.3. Cluster Differences in Achievement Goals, Motivational Types, and Affect

To evaluate whether the three clusters differed in achievement goals, motivational types, and affect, a one-way MANOVA was performed with the three clusters included as fixed factors, and other variables (four achievement goals, five motivational types, and positive and negative affect) included as dependent variables (Table 2). When the MAp, Pap, and PAv mean scores were examined, it was determined that there were statistically significantly differences between the groups (*F*_(2, 398)_ = 9.24, *p* < 0.05; partial η^2^ = 0.04; *F*_(2, 398)_ = 7.77, *p* < 0.05; partial η^2^ = 0.04; *F*_(2, 398)_ = 5.78, *p* < 0.05; partial η^2^ = 0.03, respectively). The post hoc analysis results revealed that the participants in Cluster 1 and the participants in Cluster 2 had higher scores than the participants in Cluster 3 in terms of MAp, Pap, and PAv mean scores. MAp mean scores did not differ across groups (*F*_(2, 398)_ = 2.09, *p* > 0.05). All groups had very similar scores in MAp. Regarding amotivation mean scores, the post hoc analysis results revealed that the participants in Cluster 3 had significantly higher scores than the participants in Cluster 1 (Hedges’ *g =* −0.84) and the participants in Cluster 2 (Hedges’ *g* = −0.46), and the participants in Cluster 2 had significantly higher scores than the participants in Cluster 1 (Hedges’ *g* = −0.37). There was a statistically significant difference between the groups in external regulation mean scores (*F*_(2, 398)_ = 10.36, *p* < 0.05; partial η^2^ = 0.05). The exercise participants from the moderately satisfied group (Cluster 3) had significantly higher scores than the very satisfied group (Cluster 1) and the satisfied (Cluster 2) (Hedges’ *g* = −0.63; Hedges’ *g* = −0.39; respectively). When the introjected regulation, identified regulation, intrinsic regulation, and PANAS PA mean scores were examined, there were statistically differences between the groups (*F*_(2, 398)_ = 8.38, *p* < 0.05; partial η^2^ = 0.04; *F*_(2, 398)_ = 31.09, *p* < 0.05; partial η^2^ = 0.14; *F*_(2, 398)_ = 54.03, *p* < 0.05; partial η^2^ = 0.21; *F*_(2, 398)_ = 28.82, *p* < 0.05; partial η^2^ = 0.13, respectively). The post hoc analysis results revealed that the participants in Cluster 1 had significantly higher scores than the participants in Cluster 2 and the participants in Cluster 3, and the participants in Cluster 2 had significantly higher scores than the participants in Cluster 3. Regarding PANAS NA, the participants in Cluster 3 had higher scores than the participants in Cluster 1 (Hedges’ *g* = −0.37).

## 4. Discussion

The purpose of this study was to examine achievement goals, motivation types, and affect levels according to the basic psychological need profiles of individuals in exercise environments. Based on the results of previous studies, it is assumed in this study that the level of satisfaction of the basic needs of the exercising individuals will differentiate the achievement goals, motivation types, and affect levels.

With regard to the results of achievement goals in this study, when the correlation results were examined, it was determined that three basic needs were correlated with all of the other variables at different levels, except MAv. In addition, it was also determined that individuals in the group labeled as “moderately satisfied” had lower mean scores than individuals of the other groups in all achievement goals. These results are consistent with previous research examining relationships between basic need support and achievement goals [43]. Verner-Filion et al. [44] reported that mastery- and performance-approach goals were positively associated with need satisfaction, while performance-avoidance goals were negatively associated with it. In another study, Wang and colleagues [45] determined that mastery-approach goal was positively related to the three basic needs. Some studies suggest that the satisfaction of basic psychological needs affects motivational orientations in several contexts. The basic psychological need has also been proposed to be theoretically related to achievement goals [8]. Previous research has indicated that the satisfaction of needs for autonomy and competence was associated with the adoption of mastery goals, while the satisfaction of needs for autonomy and relatedness was also associated with the adoption of performance-avoidance goals [46].

The results for contextual motivational types in this study demonstrated that adult exercisers with very satisfied basic psychological needs levels had more self-determined motivation levels. Thereby, we can say that the increase in the satisfaction level of basic needs leads to more self-determined motivation in an exercise setting. These results are in line with previous findings on basic needs and motivational regulations. Deci and Ryan [47] suggested that factors that satisfy the needs for autonomy, competence, and relatedness will promote self-determined motivation. According to SDT, when individuals’ needs for autonomy, competence, and relatedness are satisfied, they experience intrinsic motivation [8,11]. The results of the correlation between the basic needs and self-determined forms of motivation obtained in this study support this suggestion. Jõesaar and colleagues [48] determined that three basic needs were positively correlated with intrinsic motivation. Many studies (e.g., [49,50,51]) found that positive correlations existed between psychological needs and self-determined forms of motivational regulation in exercise settings. Ciani and colleagues [23] reported that the satisfaction of psychological needs for autonomy and relatedness were positively associated with autonomous motivation. Deci and Ryan [4] suggest that when the basic needs are satisfied, behaviors that may not have been initially intrinsically motivated may become internalized and be more autonomously regulated. Wilson and colleagues [52] stated that the needs for competence and autonomy positively correlated with more self-determined exercise regulations. Research by Hagger and colleagues [53] indicated that individuals with high levels of psychological need satisfaction tend to report higher levels of autonomous motivation to engage in exercise behavior. Weman-Josefsson et al. [51] reported that higher need satisfaction predicted autonomous motivation. In the same study, it was also reported that the correlations with the three basic needs and identified regulation and intrinsic motivation were moderate in size, whereas the correlation with amotivation and extrinsic regulation were weak and negative.

With regard to the results of affect in this study, this study indicated that autonomy, competence, and relatedness were positively associated with PANAS PA, while these three needs were negatively associated with PANAS NA. It also seems that PANAS PA levels were highly associated with adult exercisers feeling very satisfied about basic psychological needs. In contrast, PANAS NA levels had a low association. Also, PANAS NA scores were higher in the exercise group labeled as moderately satisfied than the exercise group labeled as the very satisfied group. These results are consistent with the literature. The results of some studies [12,54,55] demonstrated that the satisfaction of the three psychological needs positively correlated with PA and negatively correlated with NA. Similarly, Edmunds et al. [49] reported that psychological need satisfaction negatively correlated with NA. Also, a study by Kazak Çetinkalp, and Lochbaum [39] indicated that levels of psychological need satisfaction in exercise settings are related to higher levels of PA and lower levels of NA. The link between greater satisfaction of SDT-based needs with enhanced PA and reduced NA in exercise settings was supported in a study by Wilson et al. [52].

Limitations of the present study include the lack of studies in the exercise environment assessing the relationships among variables in this study. Exercise participants joining in various exercise groups at different exercise levels and ranges of age were included in the present study. The variables might be examined more consistently in group partaking in a single type of exercise and at a more specific age range. Research examining gender differences could provide a different perspective too. Future research should also reexamine relationships between achievement goals, motivational levels, and affect levels, and basic psychological needs considering age, exercise groups, and gender in order to more objectively identify which clusters lead to more adaptive/maladaptive outcomes. Also, analyzing basic psychological needs with the effects of age, gender, and exercise type could also enable researchers to gain deeper insights into the issue. Finally, the investigation of the relationships in different cultures may reveal cultural differences.

## 5. Conclusions

The current study identified a number of interrelations between basic need satisfaction and achievement goals, affect, and motivational types among participants in an exercise setting. Even with limitations and future research considerations, overall, this study showed that satisfying basic psychological needs in the exercise environment is essential. Clearly, the above-mentioned motivational variables are important for individuals’ participation in exercise settings. It is necessary to keep in mind that the satisfaction of these needs leads to more positive behavioral outcomes. These findings may be useful for further theoretical development as regards trajectories of basic need support in terms of achievement goals, affect, and motivational types.

## Figures and Tables

**Table 1:** Descriptive statistics and correlations between all variables of the overall sample.

	*M*	*SD*	1	2	3	4	5	6	7	8	9	10	11	12	13	14
1. MAp	5.52	1.55	1													
2. MAv	3.69	1.66	0.21 **	1												
3. PAp	4.36	1.83	0.45 **	0.43 **	1											
4. PAv	3.87	1.75	0.32 **	0.58 **	0.78 **	1										
5. Amotivation	1.28	0.42	−0.21 **	0.06	−0.04	0.03	1									
6. External Regulation	1.42	0.49	−0.12 *	0.11 *	0.05	0.13 *	0.53 **	1								
7. Introjected regulation	3.12	1.04	0.16 **	0.09	0.28 **	0.23 **	−0.08	0.02	1							
8. Identified Regulation	4.20	0.61	0.24 **	0.07	0.24 **	0.19 **	−0.39 **	−0.18 **	0.57 **	1						
9. Intrinsic Regulation	4.39	0.61	0.28 **	0.09	0.24 **	0.17 **	−0.41 **	−0.31 **	0.34 **	0.64 **	1					
10. PANAS PA	36.14	6.72	0.21 **	0.02	0.16 **	0.12 *	−0.19 **	−0.13 **	0.20 **	0.31 **	0.28 **	1				
11. PANAS NA	18.07	6.15	−0.16 **	0.16 **	0.08	0.06	0.11 *	0.07	0.03	−0.08	−0.06	−0.16 **	1			
12. Autonomy	4.13	0.56	0.21 **	0.09	0.20 **	0.18 **	−0.32 **	−0.17 **	0.23 **	0.41 **	0.50 **	0.35 **	−0.19 **	1		
13. Competence	4.14	0.57	0.22 **	0.06	0.19 **	0.16 **	−0.30 **	−0.25 **	0.23 **	0.44 **	0.49 **	0.43 **	−0.19 **	0.81 **	1	
14. Relatedness	4.06	0.63	0.17 **	0.04	0.11 *	0.11 *	−0.25 **	−0.16 **	0.16 **	0.29 **	0.38 **	0.36 **	−0.19 **	0.73 **	0.71 **	1

Note: * *p* < 0.05 ** *p* < 0.01; *M*: Mean; *SD*: Standard deviation; MAp: mastery-approach; MAv: mastery-avoidance; PAp: performance-approach; PAv: performance-avoidance; PANAS: Positive and Negative Affect Schedule; PA: positive affect; NA: negative effect.

**Table 2:** Mean and standard deviation values according to the clusters and the results of multivariate analysis of variance.

	Cluster 1 Very Satisfied (*n* = 123)	Cluster 2 Satisfied (*n* = 194)	Cluster 3 Moderately Satisfied (*n* = 84)				Hedges’ *g*		
Variable	*M* (*SD*)	*M* (*SD*)	*M* (*SD*)	*F*_(2, 398)_	$\eta_{p}^{2}$	Post hoc	1–2	1–3	2–3
MAp	5.88 (1.63)	5.54 (1.47)	4.96 (1.47)	9.24	0.04	3 < 1, 2	0.22	0.59	0.39
MAv	3.76 (1.93)	3.79 (1.55)	3.36 (1.46)	2.09	0.01	None	---	---	---
PAp	4.66 (1.99)	4.46 (1.76)	3.69 (1.61)	7.77	0.04	3 < 1, 2	---	0.52	0.45
PAv	4.09 (2.03)	3.98 (1.62)	3.31 (1.46)	5.78	0.03	3 < 1, 2	---	0.43	0.43
Amotivation	1.14 (0.33)	1.28 (0.41)	1.48 (0.49)	17.46	0.08	1 < 2 < 3	−0.37	−0.84	−0.46
External Regulation	1.29 (0.44)	1.41 (0.47)	1.61 (0.59)	10.36	0.05	1, 2 < 3	---	−0.63	−0.39
Introjected Regulation	3.38 (1.21)	3.11 (0.99)	2.79 (0.78)	8.38	0.04	3 < 2 < 1	0.25	0.56	0.34
Identified Regulation	4.46 (0.57)	4.21 (0.54)	3.82 (0.63)	31.09	0.14	3 < 2 < 1	0.45	1.07	0.68
Intrinsic Regulation	4.72 (0.43)	4.37 (0.56)	3.93 (0.64)	54.03	0.21	3 < 2 < 1	0.68	1.50	0.75
PANAS PA	38.94 (6.15)	36.07 (6.34)	32.18 (6.41)	28.82	0.13	3 < 2 < 1	0.46	1.08	0.61
PANAS NA	16.86 (5.37)	18.35 (6.01)	19.18 (7.25)	3.99	0.02	1 < 3	---	−0.37	---

Note. MAp: mastery-approach; MAv: mastery-avoidance; PAp: performance-approach; PAv: performance-avoidance; PANAS: Positive and Negative Affect Schedule; PA: positive affect; NA: negative effect; *M*: Mean; *SD*: Standard deviation; $\eta_{p}^{2}$: Partial eta squared.
